# Supplementary material for: Co-Existence of Iron Oxide Nanoparticles and Manganese Oxide Nanorods as Decoration of Hollow Carbon Spheres for Boosting Electrochemical Performance of Li-Ion Battery
Source: Materials (Basel). 2021 Nov 15;14(22):6902. doi: 10.3390/ma14226902 (PMC8620810; doi:10.3390/ma14226902)
Supplement: Supplementary file 1 [file materials-14-06902-s001.zip › materials-1399479-supplementary.pdf]

materials

MDPI

Supplementary Materials

Co-Existence of Iron Oxide Nanoparticles and Manganese Oxide Nanorods as Decoration of Hollow Carbon Spheres for Boosting Electrochemical Performance of Li-Ion Battery

Karolina Wenelska *, Martyna Trukawka, Wojciech Kukulka, Xuecheng Chen and Ewa Mijowska

Department of Nanomaterials Physicochemistry, Faculty of Chemical Technology and Engineering, West Pomeranian University of Technology, Szczecin, Piastow Ave. 42, 71-065 Szczecin, Poland; mtrukawka@zut.edu.pl (M.T.); wkukulka@zut.edu.pl (W.K.); xchen@zut.edu.pl (X.C.); emijowska@zut.edu.pl (E.M.)
* Correspondence: kwenelska@zut.edu.pl

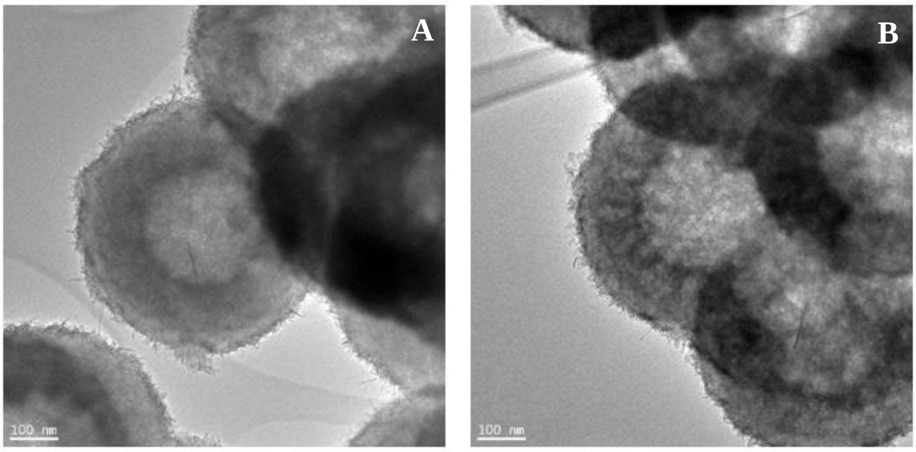

Figure S1. Transmission electron microscopy (**A**,**B**) images of MnO_2 rods on the hollow carbon spheres surface.

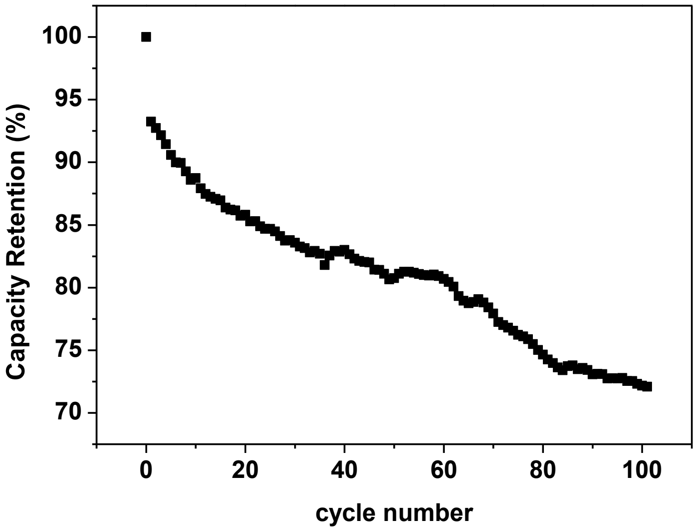

Figure S2. Cycling stability at 100 mA/g.
